# LncRNA XIST regulates atherosclerosis progression in ox-LDL-induced HUVECs

**DOI:** 10.1515/med-2021-0200

**Published:** 2021-01-08

**Authors:** Hongmei Gao, Zhaohui Guo

**Affiliations:** Department of Neurology, The Fourth Affiliated Hospital of Harbin Medical University, No. 37 Yiyuan Street, Nangang District, 150001, Harbin, Heilongjiang, China

**Keywords:** XIST, miR-98-5p, PAPPA, ox-LDL, HUVECs

## Abstract

Long noncoding RNAs (lncRNAs) have been verified as vital regulators in human disease, including atherosclerosis. However, the precise role of X-inactive-specific transcript (XIST) in atherosclerosis remains unclear. The proliferation and apoptosis of human umbilical vein endothelial cells (HUVECs) exposed to low-density lipoprotein (ox-LDL) were assessed by 3-(4,5-dimethylthiazol-2-yl)-2,5-diphenyl-2*H*-tetrazol-3-ium bromide, and flow cytometry assays, correspondingly. The western blot assay was used to quantify protein expression. Lactate dehydrogenase activity and the concentrations of inflammatory factors were measured by matched kits. The real-time quantitative polymerase chain reaction (qPCR) was used to determine α-smooth muscle actin, smooth muscle protein 22-α, XIST, miR-98-5p, and pregnancy-associated plasma protein A (PAPPA) levels in HUVECs. The relationship among XIST, miR-98-5p, and PAPPA was analyzed by dual-luciferase reporter, RNA immunoprecipitation, and RNA pull-down assays. We found ox-LDL repressed proliferation and induced inflammation and apoptosis in HUVECs. Loss-of-functional experiment suggested that the downregulation of XIST overturned the ox-LDL-induced effects on HUVECs. Additionally, overexpression of miR-98-5p-induced effects on ox-LDL-stimulated HUVECs was abolished by upregulation of XIST. However, silencing of miR-98-5p strengthened the ox-LDL-induced effects on HUVECs by increasing expression of PAPPA. Mechanistically, XIST could regulate PAPPA expression in ox-LDL-induced HUVECs by sponging miR-98-5p, providing understanding for atherosclerosis.

## 1 Introduction

Atherosclerosis is the most common cause of cerebrovascular disease and is a complex polygenic disease with high mortality [1], while its pathogenesis has not been fully understood [2]. Oxidized low-density lipoprotein (ox-LDL) is a foremost inducer for atherosclerosis [3]. Ox-LDL could enhance proliferation and invasion of vascular smooth muscle cells (VSMCs) [4]; meanwhile, several investigations have demonstrated that ox-LDL promoted the apoptosis of human umbilical vein endothelial cells (HUVECs) and regulated the activity of caspase 3 and caspase 9. Conclusively, ox-LDL played critical roles in the pathogenesis of cerebrovascular disease [5,6].

Although noncoding property, long noncoding RNAs (lncRNAs), more than 200 nucleotides in length, were implicated in numerous biological behaviors by regulation of gene expression [7]. Moreover, accumulating evidence suggested the lncRNAs played key roles in atherosclerosis, implicating proliferation and apoptosis of smooth muscle and endothelial cells, inflammation, and lipid metabolism [8,9]. Increasing evidence has confirmed that XIST was increased in various human diseases, including nasopharyngeal carcinoma [10], glioblastoma [11], and lung cancer [12]. Additionally, XIST was overexpressed in human brain microvascular endothelial cells exposed to hypoxia [13]. Nevertheless, the regulatory mechanism of XIST was unidentified in atherosclerosis.

MiRNAs, noncoding RNAs and key mediators in diverse biological pathways, could control cellular activity under pathologic situations, including atherosclerosis [14]. Therefore, mechanistic dissection of miRNA functions may help us to comprehend the roles of miRNAs in disease, which promoted the development of miRNA-based therapeutics [15]. Sun et al. and Li et al. confirmed that miR-98 protected myocardium cells or endothelial cells against myocardial infarction or hypoxia/reoxygenation-induced apoptosis by targeting caspase-3, respectively, indicating that miRNA-98 was a stress-specific miRNA participating in cell survival and apoptosis [16,17]. However, the mechanism of miR-98-5p in ox-LDL-stimulated HUVECs remains unknown.

Pregnancy-associated plasma protein A (PAPPA), a zinc metalloproteinase, played significant roles in atherosclerosis [18]. The elevated PAPPA level was a poor prognosis factor in vascular diseases [19]. Therefore, the role of PAPPA in atherosclerosis was worth investigating. In this study, the influences of ox-LDL in proliferation, inflammation, and apoptosis of HUVECs, as well as XIST, miR-98-5p, and PAPPA in HUVECs under ox-LDL administration, were investigated.

## 2 Materials and methods

### 2.1 Cell lines and cell culture

HUVECs were bought from American Type Culture Collection (Rockville, MD, USA) and cultured in DMEM medium (GIBCO BRL, Grand Island, NY, USA) supplemented with 10% fetal bovine serum (FBS; GIBCO BRL), 100 μg/mL of streptomycin, and 100 U/mL of penicillin (GIBCO BRL). Cells were cultivated in standard culture conditions (5% CO_2_, 37°C). Ox-LDL (Sigma, St. Louis, MO, USA) was used to culture cells to establish an arteriosclerosis stimulation for HUVECs. The usage of HUVECs was approved by the Ethics Committee of Fourth Affiliated Hospital of Harbin Medical University.

### 2.2 3-(4,5-Dimethylthiazol-2-yl)-2,5-diphenyl-2*H*-tetrazol-3-ium bromide (MTT) assay

Cell viability was tested by MTT assay. In brief, 2.0 × 10^3^ HUVECs were sowed into 96 wells and allowed to adhere. After incubation for indicated times, 10 μL of 5 mg/mL MTT (Beyotime, Jiangsu, China) solution was added to each well of the 96 wells plate and allowed to incubate for additional 4 h. The supernatant was replaced by 150 μL of dimethyl sulfoxide (DMSO) and then shocked for 10 min. The absorbance was detected at 490 nm under a microplate spectrophotometer (Olympus, Tokyo, Japan).

### 2.3 Cell apoptosis assay

Annexin V labeled with fluorescein isothiocyanate (FITC)/propidium iodide (PI) Apoptosis Detection Kit (Thermo Fisher Scientific, Waltham, MA, USA) was performed for cell apoptosis assay. After treatment, HUVECs were harvested by trypsinization. Then, cells were resuspended with phosphate buffer saline buffer solution into a single cell suspension (1 × 10^7^/mL). 100 μL of cell suspension was reacted with 5 μL of Annexin V-FITC and 5 μL of PI in the dark condition. The apoptotic cells were measured using flow cytometer (Applied Biosystems, Foster City, CA, USA) and the Flowjo V10 software (Tree Star, San Francisco, CA, USA) was used to quantify the results.

### 2.4 Western blot assay

Radio-immunoprecipitation assay buffer (Beyotime) containing protease inhibitors was used to extract proteins from HUVECs at 4°C. In addition, protein concentrations were checked with a bicinchoninic acid (BCA) protein assay (Solarbio, Beijing, China). Proteins were fractionated on 12% sodium dodecyl sulfate polyacrylamide gel electrophoresis, and then electroblotted onto the polyvinylidene difluoride membranes (GE Healthcare, Piscataway, NJ, USA). After being blocked with 3% Albumin Bovine V (Amyjet scientific, Wuhan, China), the membranes were interacted with the indicated antibodies. After being washed, membranes were incubated with the Goat polyclonal Secondary Antibody to Rabbit IgG-H&L (ab150077; 1:2,000 dilution; Abcam, Cambridge, MA, USA) for 2 h. Eventually, the western blot bands were visualized by ECL Western Blotting Detection Kit (Solarbio) under Alpha Innotech Imaging System (Protein Simple, Santa Clara, CA, USA). The related protein expression was standardized to β-actin. The primary antibodies were listed: anti-BCL2-Associated X (Bax; ab32503; 1:1,000 dilution; Abcam), anti-B-cell lymphoma-2 (Bcl-2; ab32124; 1:1,000 dilution; Abcam), anti-α-smooth muscle actin (α-SMA; ab5694; 1:1,000 dilution; Abcam), anti-smooth muscle protein 22-α (SM22-α; ab14106; 1:1,000 dilution; Abcam), anti-PAPPA (ab174314; 1:1,000 dilution; Abcam), and β-actin (ab179467; 1:3,000 dilution; Abcam).

### 2.5 Measurement of lactate dehydrogenase (LDH)

Cytotoxicity was evaluated by measuring LDH released in the medium after treatment with ox-LDL at different time points using the LDH activity detection kit (Solarbio) as described by Wang et al. [20].

### 2.6 Enzyme-linked immunosorbent assay (ELISA)

The concentrations of interleukin 6 (IL-6), interleukin 1β (IL-1β), and tumor necrosis factor α (TNF-α) in medium were evaluated by IL-6 ELISA KIT, IL-1β ELISA KIT, and TNF-α ELISA KIT (Boster, Wuhan, China) as instructed by the manufacturer, respectively. The absorbance was measured under a microplate spectrophotometer (Olympus).

### 2.7 Real-time quantitative polymerase chain reaction (qPCR)

Total RNA was isolated using TriQuick Reagent (Solarbio) in accordance with the producer’s procedures. For lncRNA/mRNA, RNA was used to synthesize complementary DNA (cDNA) with a cDNA Reverse Transcription kit (Bio-Rad, Hercules, CA, USA). The expression levels of lncRNA/mRNA were evaluated using Quantitect SYBR Green Kit (Qiagen, Hilden, Germany) based on the 2^−ΔΔCt^ method, with glyceraldehyde-3-phosphate dehydrogenase (GAPDH) as an internal control. For miR-98-5p, cDNA was synthesized and amplified using miScript II RT kit (Qiagen) under Thermal Cycler CFX6 System (Bio-Rad). The expression level of miR-98-5p was standardized to endogenous small nuclear RNA U6.

The sequences of primers used were listed:

α-SMA (Forward-5ʹ-GTCCACCGCAAATGCTTCTAA-3ʹ; Reverse-5ʹ- AAAACACATTAACGAGTCAG-3ʹ);
SM22-α (Forward-5ʹ-TGATTCTGAGCAAGCTGGT-3ʹ; Reverse-5ʹ- TGCCTTCAAAGAGGTCAAC -3ʹ);
XIST (Forward-5ʹ-CTCTCCATTGGGTTCAC-3ʹ; Reverse-5ʹ- GCGGCAGGTCTTAAGAGATGAG -3ʹ);
miR-98-5p (Forward-5ʹ-GCCGAGTGAGGUAGTAAGTTG-3ʹ; Reverse-5ʹ- CTCAACTGGTGTCGTGGA-3ʹ);
PAPPA (Forward-5ʹ-ACAAAGACCCACGCTACTTTTT-3ʹ; Reverse-5ʹ- CATGAACTGCCCATCATAGGTG-3ʹ);
GAPDH (Forward-5ʹ-TCCCATCACCATCTTCCAGG-3ʹ; Reverse-5ʹ- GATGACCCTTTTGGCTCCC-3ʹ);
U6 (Forward-5ʹ-AACGCTTCACGAATTTGCGT-3ʹ; Reverse-5ʹ- CTCGCTTCGGCAGCACA-3ʹ).

### 2.8 Transfection assay

Small interfering RNA (siRNA) against XIST (si-XIST) or PAPPA (si-PAPPA) and siRNA scrambled control (si-NC), XIST overexpression vector (pcDNA-XIST), and its negative control (pcDNA) were acquired from GenePharma (Shanghai, China). MiR-98-5p mimic (miR-98-5p) and control (miR-NC) and miR-98-5p inhibitor (anti-miR-98-5p) and control (anti-NC) were provided by Sangon (Shanghai, China). Different oligonucleotides or vectors were transfected into HUVECs with Lipofectamine 2000 reagent (Qiagen) following the user’s guidebook.

### 2.9 Dual-luciferase reporter assay

We predicted the miR-98-5p binding sites in XIST or 3ʹuntranslated region (UTR) of PAPPA using the bioinformatics database Starbase3.0 (http://starbase.sysu.edu.cn/). For the generation of XIST-WT, XIST-MUT, PAPPA 3ʹUTR-WT, or PAPPA 3ʹUTR-MUT report vectors, the WT or MUT fragment of XIST and PAPPA 3ʹUTR were subcloned into the pGL3 vectors (Realgene, Nanjing, China). For the luciferase activity measurement, HUVECs were co-transfected with constructed luciferase reporter vectors according to the experiment design and miR-98-5p mimic or miR-NC. After 48 h, luciferase activity was detected using the Dual-Luciferase Assay Kit (GeneCopoeia, Rockville, MD, USA).

### 2.10 RNA immunoprecipitation (RIP) assay

The RIP assay was carried out with the Magna RIP RNA-Binding Protein Immunoprecipitation Kit (Sigma) according to manufacturer’s instruction. Magnetic beads were pre-conjugated with antibody against Argonaute2 (Ago2; Millipore, Bedford, MA, USA), with IgG as control. HUVECs were collected and then lysed by RIP-buffer, and the lysates were treated with bead antibody complex. After incubation at 4°C overnight, immunoprecipitated RNA was treated with proteinase K buffer and then reverse-transcribed. The abundances of miR-98-5p, PAPPA, and XIST were assessed by qPCR analysis.

### 2.11 RNA pull-down

The biotin-labeled Bio-miR-98-5p and Bio-NC were synthesized by RiboBio (Guangzhou, China). In brief, 1 × 10^7^ HUVECs were infected with Bio-miR-98-5p or Bio-NC at a final concentration of 50 nM. After 48 h, HUVECs were lysed and incubated with magnetic beads coupled with streptavidin (Life Technologies, Carlsbad, CA, USA). After pulling down, biotin-coupled RNA complex was purified by proteinase K and subjected to qPCR assay.

### 2.12 Statistical analysis

The statistics software SPSS 21.0 software (IBM, Somers, NY, USA) was conducted for statistical analysis. The significant differences in selected two groups were determined by Student’s *t*-test, while differences of multigroups were analyzed by one-way analysis of variance (ANOVA) with Turkey’s test. *P* < 0.05 was indicated significant differences, and the data were exhibited as mean ± standard deviation.

## 3 Results

### 3.1 Ox-LDL inhibited proliferation and induced apoptosis and inflammatory response in HUVECs

**Figure 1:**
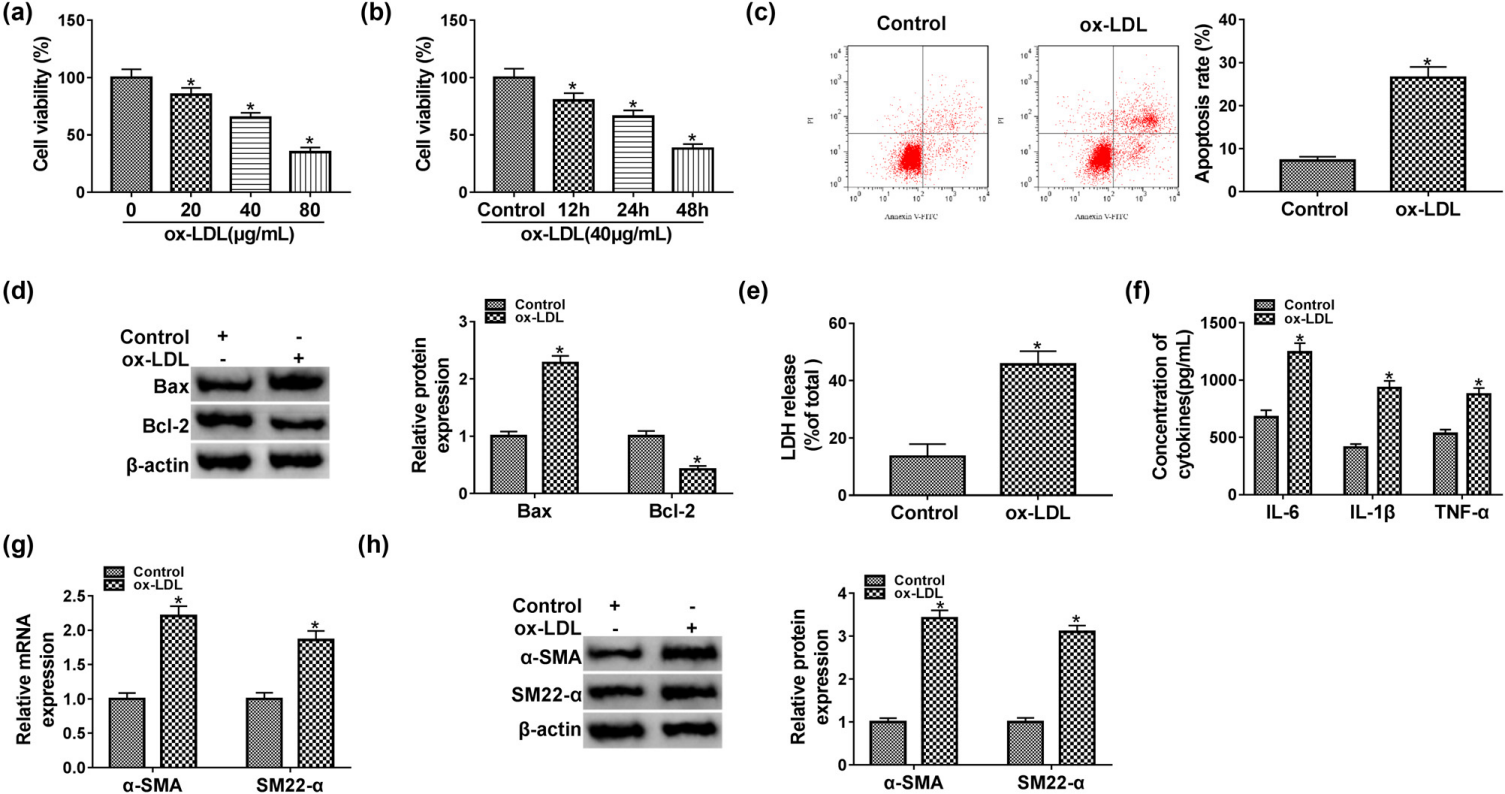
Ox-LDL suppressed proliferation and enhanced apoptosis and inflammatory response in HUVECs. (a and b) The cell viability of HUVECs exposed to ox-LDL was assessed by MTT assay. (c) The flow cytometry assay was performed for examining the apoptosis rate of HUVECs treated with 40 μg/mL of ox-LDL for 48 h. (d) The protein expression levels of Bax and Bcl-2 were measured by western blot assay. (e) LDH release in the medium was examined by a commercial kit. (f) The levels of IL-6, IL-1β, and TNF-α were evaluated in the medium by matched kits. (g and h) The qPCR and western blot assays were used to show the expression levels of α-SMA and SM22-α in HUVECs treated with ox-LDL. Data shown are mean ± SD and from three independent experiments. *P < 0.05. Abbreviations: oxidized low-density lipoprotein (ox-LDL), anti-BCL2-Associated X (Bax), anti-B-cell lymphoma-2 (Bcl-2), interleukin 6 (IL-6), interleukin 1β (IL-1β), and tumor necrosis factor α (TNF-α), anti-α-smooth muscle actin (α-SMA), anti-smooth muscle protein 22-α (SM22-α).

Originally, cell viability of HUVECs was assessed by MTT in HUVECs, and the results indicated that cell viability of HUVECs was declined by ox-LDL in dose/time-dependent manner (Figure 1a and b). Furthermore, apoptosis rate was significantly higher in HUVECs treated with 40 μg/mL of ox-LDL for 48 h compared with control group (Figure 1c). We also measured the expression levels of Bax and Bcl-2 in HUVECs by western blot analysis, and the results revealed that Bax protein level was upregulated, while Bcl-2 was downregulated in HUVECs treated with ox-LDL when compared with control group (Figure 1d). After treatment with 40 μg/mL of ox-LDL for 48 h, the ox-LDL group had the higher LDH release compared with control group (Figure 1e). In ox-LDL condition, inflammatory response was enhanced in HUVECs by upregulating release of inflammatory factors, including IL-6, IL-1β, and TNF-α (Figure 1f). In addition, the mRNA and protein expression levels of α-SMA and SM22-α were upregulated in HUVECs exposed to ox-LDL (Figure 1g and h). In summary, Ox-LDL promoted inflammatory response and apoptosis, as well as suppressed proliferation in HUVECs.

### 3.2 Knockdown of XIST abolished the effects of ox-LDL on proliferation, apoptosis, and inflammatory response of HUVECs

**Figure 2:**
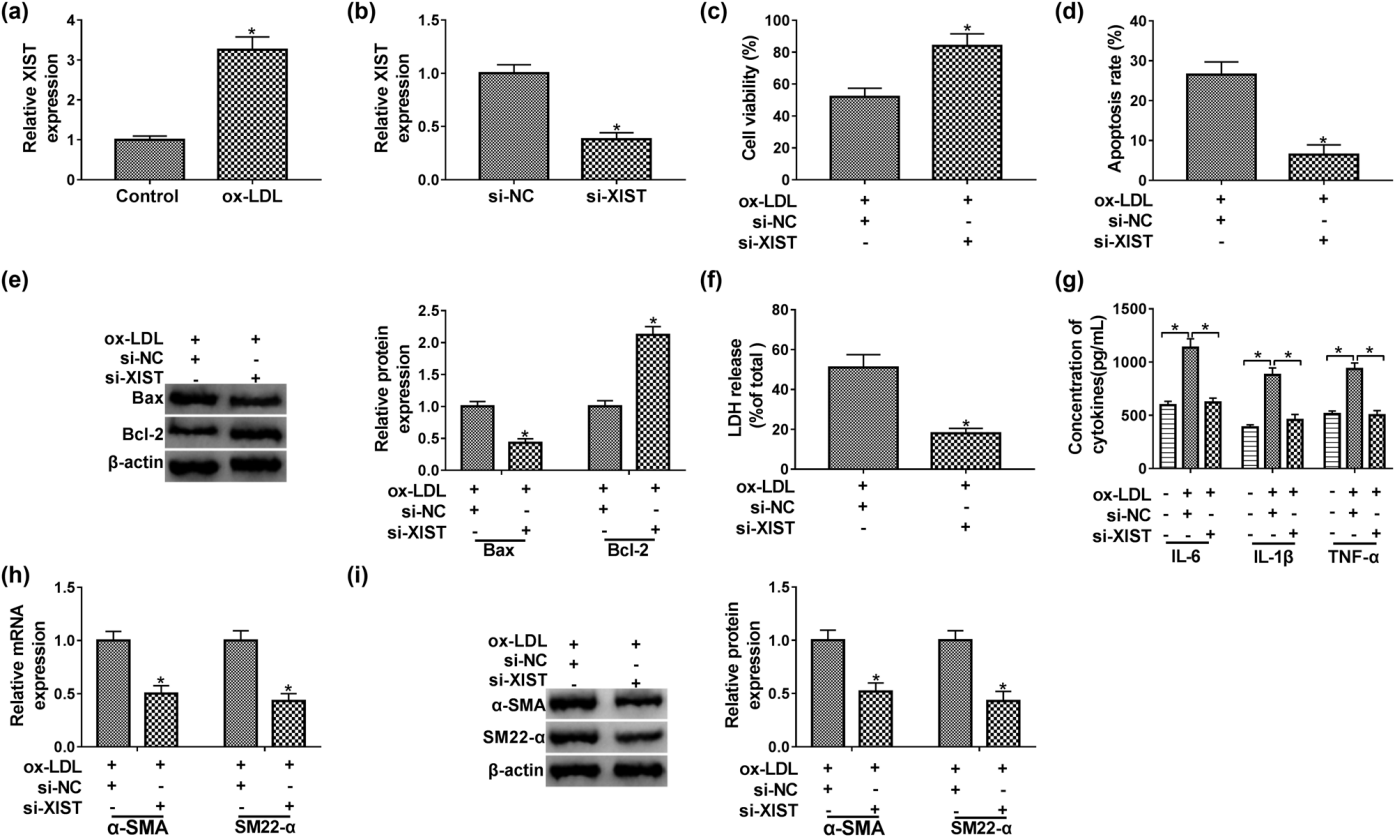
Knockdown of XIST weakened ox-LDL-induced effects on HUVECs. (a) The expression level of XIST in HUVECs treated with ox-LDL was determined by qPCR analysis. (b) The interference efficiency of si-XIST in HUVECs was assessed by qPCR assay. (c–f) HUVECs were divided into two groups: ox-LDL + si-NC and ox-LDL + si-XIST groups. (c) The proliferation capability of HUVECs was measured using MTT assay. (d) The flow cytometry assay was recruited to monitor the apoptosis of HUVECs. (e) The western blot analysis was applied to assess Bax and Bcl-2 expression in HUVECs. (f) A commercial available kit was used to assess LDH release in HUVECs supernatants. (g) The expression levels of IL-6, IL-1β, and TNF-α in ox-LDL-induced HUVECs were displayed, with 0 μg/mL of ox-LDL group as control. (h and i) QPCR and western blot analyses were employed to quantify mRNA and protein levels of α-SMA and SM22-α in HUVECs, respectively. Data shown are mean ± SD and from three independent experiments. *P < 0.05. Abbreviations: X-inactive-specific transcript (XIST).

By performing qPCR analysis, we noticed that ox-LDL induced the upregulation of XIST in HUVECs (Figure 2a). The expression level of XIST was decreased in the si-XIST transfected cells than those cells transfected with si-NC (Figure 2b). As presented in Figure 2c, cell viability was increased in ox-LDL-induced HUVECs after knockdown of XIST. In contrast, under ox-LDL condition, HUVECs transfected with si-XIST exhibited fewer apoptotic cells than that in si-NC group (Figure 2d). The western blot analysis revealed that si-XIST significantly decreased the Bax expression, while upregulated Bcl-2 expression in ox-LDL-induced HUVECs (Figure 2e). LDH release was declined in HUVECs treated with si-XIST and ox-LDL than those cells treated with si-NC and ox-LDL (Figure 2f). Moreover, knockdown of XIST significantly weakened inflammatory response by declining release of IL-6, IL-1β, and TNF-α in ox-LDL-induced HUVECs (Figure 2g). Additionally, silencing of XIST obviously decreased the expression levels of α-SMA and SM22-α in HUVECs treated with ox-LDL (Figure 2h and i). These results suggested that ox-LDL regulated proliferation, apoptosis, and inflammatory response of HUVECs by upregulating XIST expression.

### 3.3 XIST negatively regulated miR-98-5p expression in HUVECs

**Figure 3:**
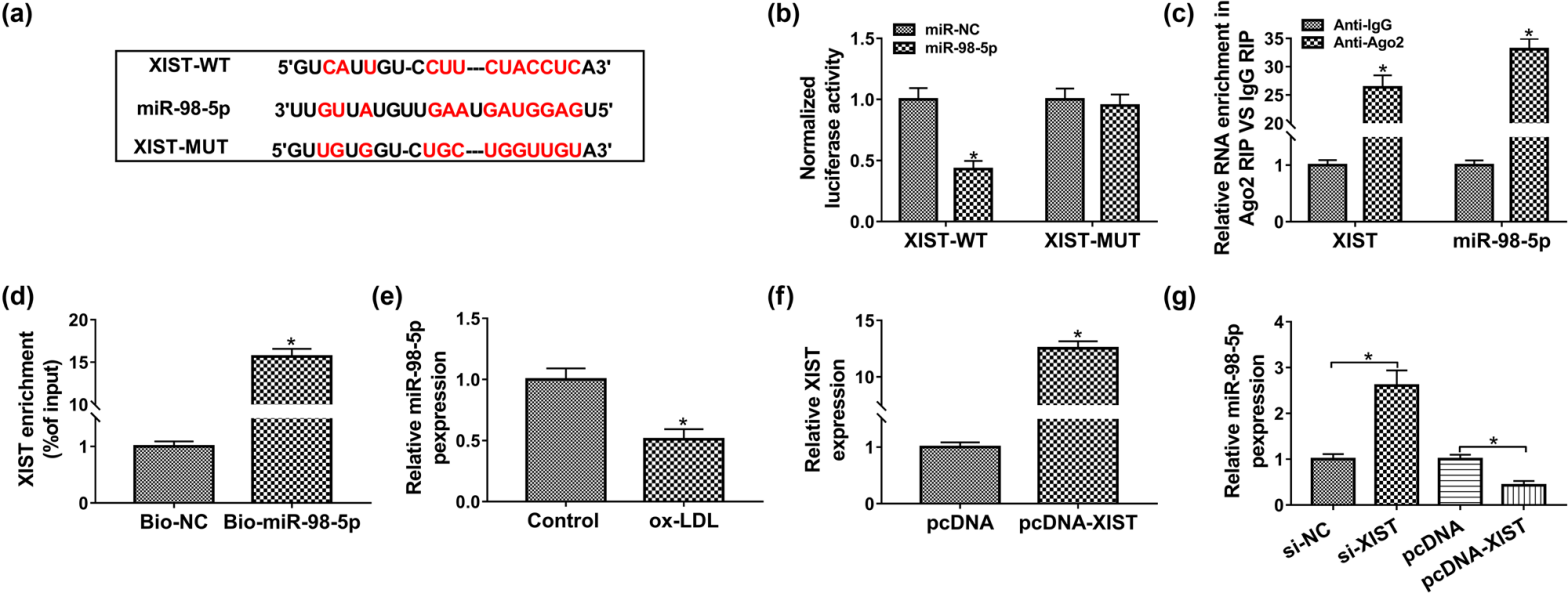
MiR-98-5p was a direct target of XIST. (a) Binding region between miR-98-5p and XIST was predicted by bioinformatics software. (b) Dual-luciferase reporter assay was performed in HUVECs to measure the luciferase activity. (c) After RIP assay, qPCR assay was conducted to assess the enrichments of miR-98-5p and XIST in cell lysates. (d) RNA pull-down assay was used to confirm the association between miR-98-5p and XIST. (e) The expression level of miR-98-5p in HUVECs exposed to ox-LDL was determined by qPCR assay. (f) The transfection efficiency of pcDNA-XIST in HUVECs was checked by qPCR assay. (g) The expression level of miR-98-5p was assessed by qPCR assay in HUVECs transfected with si-NC, si-XIST, pcDNA, or pcDNA-XIST. Data shown are mean ± SD and from three independent experiments. *P < 0.05.

Bioinformatics database Starbase3.0 was used to predict the potential target of XIST. Binding regions between XIST and miR-98-5p, as well as matched mutant sites of XIST-MUT, were displayed in Figure 3a. In addition, the luciferase activity of XIST-WT group was decreased under miR-98-5p overexpression, while XIST-MUT showed resistance to the impact of miR-98-5p overexpression in HUVECs (Figure 3b). And we also found that Ago2 antibody dramatically enriched XIST and miR-98-5p levels compared to the IgG control by RIP analysis (Figure 3c). After RNA pull-down assay, we noticed that XIST was remarkably enriched in the Bio-miR-98-5p group in comparison with control group (Figure 3d). Moreover, ox-LDL repressed the expression of miR-98-5p in HUVECs (Figure 3e). After transfection with overexpression vector of XIST into HUVECs, we found that XIST was significantly higher in HUVECs transfected with pcDNA-XIST compared with cells transfected with pcDNA (Figure 3f). The functional experiments indicated that silencing of XIST enhanced the expression of miR-98-5p, while overexpression of XIST resulted in opposite results in HUVECs (Figure 3g). Conclusively, XIST regulated miR-98-5p expression in a negative feedback mode in HUVECs exposed to ox-LDL.

### 3.4 Overexpression of XIST abrogated the upregulation of miR-98-5p-induced effects on proliferation, apoptosis, and inflammatory response in ox-LDL-induced HUVECs

**Figure 4:**
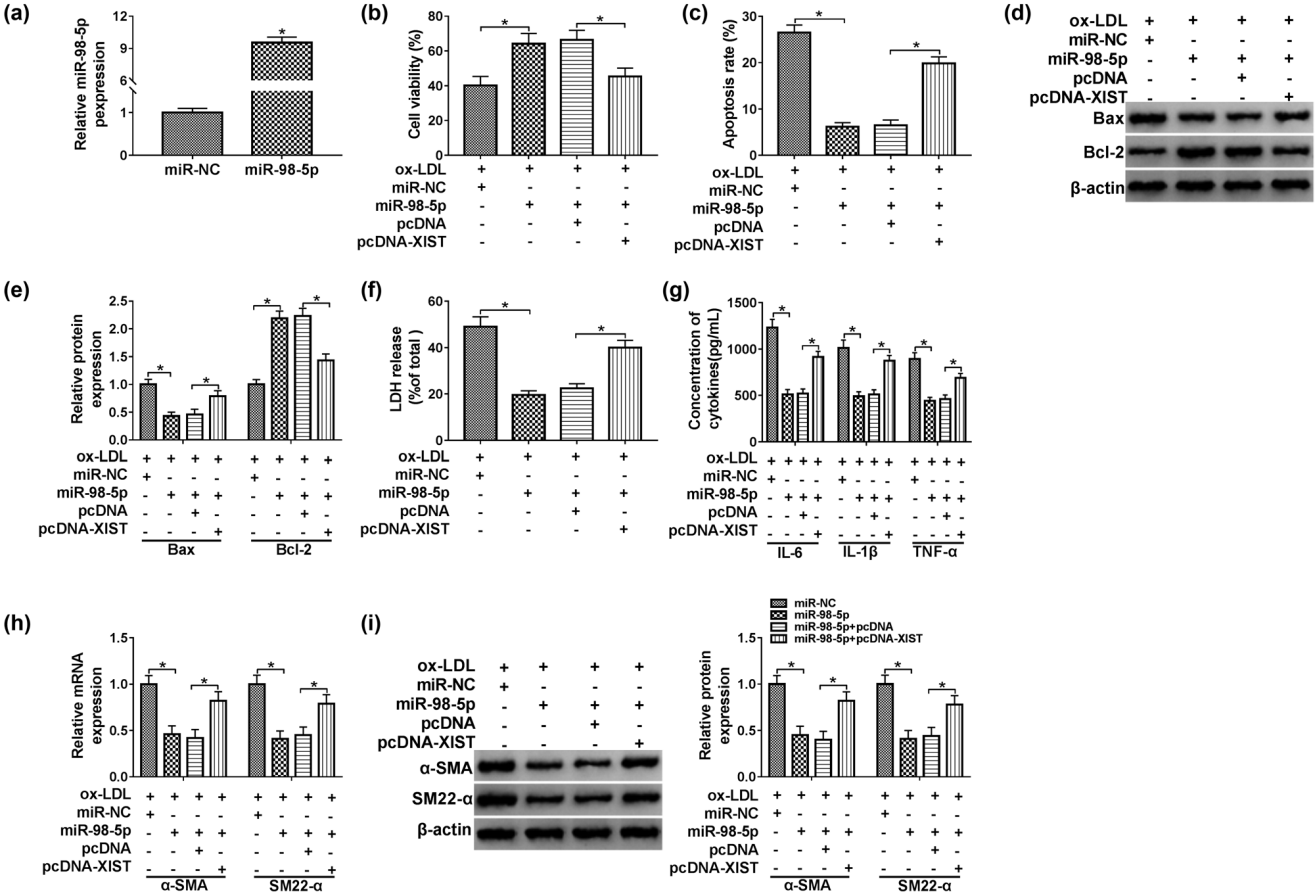
Ox-LDL repressed proliferation and induced apoptosis and inflammatory response in HUVECs by targeting the XIST/miR-98-5p axis. (a) QPCR was enforced to confirm the overexpression efficiency of miR-98-5p in HUVECs. (b–i) HUVECs were treated with ox-LDL + miR-NC, ox-LDL + miR-98-5p, ox-LDL + miR-98-5p + pcDNA, or ox-LDL + miR-98-5p + pcDNA-XIST. (b) MTT assay was used to assess cell viability of HUVECs. (c) Cells apoptosis assay was performed in transfected HUVECs by flow cytometry assay. (d and e) The protein expression levels of Bax and Bcl-2 were calculated in HUVECs by western blot assay. (f) The LDH release was displayed in the different groups by kit assay. (g) The concentrations of IL-6, IL-1β, and TNF-α in the medium were presented by ELISA assay. (h and i) The mRNA and protein expression levels of α-SMA and SM22-α in HUVECs were detected by qPCR and western blot assays, respectively. Data shown are mean ± SD and from three independent experiments. *P < 0.05.

As above results had confirmed that miR-98-5p was a target of XIST, the association between miR-98-5p and XIST was investigated. MiR-98-5p was overexpressed in HUVECs transfected with miR-98-5p compared with miR-NC group (Figure 4a). The results of MTT analysis revealed that upregulation of XIST weakened the enhancement effect on cell proliferation induced by overexpression of miR-98-5p when HUVECs were exposed to ox-LDL (Figure 4b). Reduction of apoptosis rate induced by miR-98-5p overexpression could be resumed by transfection with pcDNA-XIST into ox-LDL-induced HUVECs (Figure 4c). Western blot assay implied that overexpression of miR-98-5p inhibited Bax, while enhanced Bcl-2 expression in HUVECs treated with ox-LDL, which was overturned by co-transfection with pcDNA-XIST (Figure 4d and e). Next, we performed LDH assay and found that overexpression of miR-98-5p significantly suppressed the LDH release in HUVECs exposed to ox-LDL, which was counteracted by the upregulation of XIST (Figure 4f). The inhibitory effects on IL-6, IL-1β, and TNF-α expression in ox-LDL-induced cells caused by miR-98-5p mimic were reversed by overexpression of XIST (Figure 4g). Furthermore, upregulation of miR-98-5p impeded the expression of α-SMA and SM22-α, including mRNA and protein, whereas this effect could be abolished by ectopic expression of XIST in ox-LDL-induced HUVECs (Figure 4h and i). Collectively, XIST could strengthen the ox-LDL-induced enhancement effects on apoptosis and inflammatory response, as well as inhibitory effect on proliferation in HUVECs by regulating miR-98-5p.

### 3.5 PAPPA was a target gene of miR-98-5p in HUVECs

**Figure 5:**
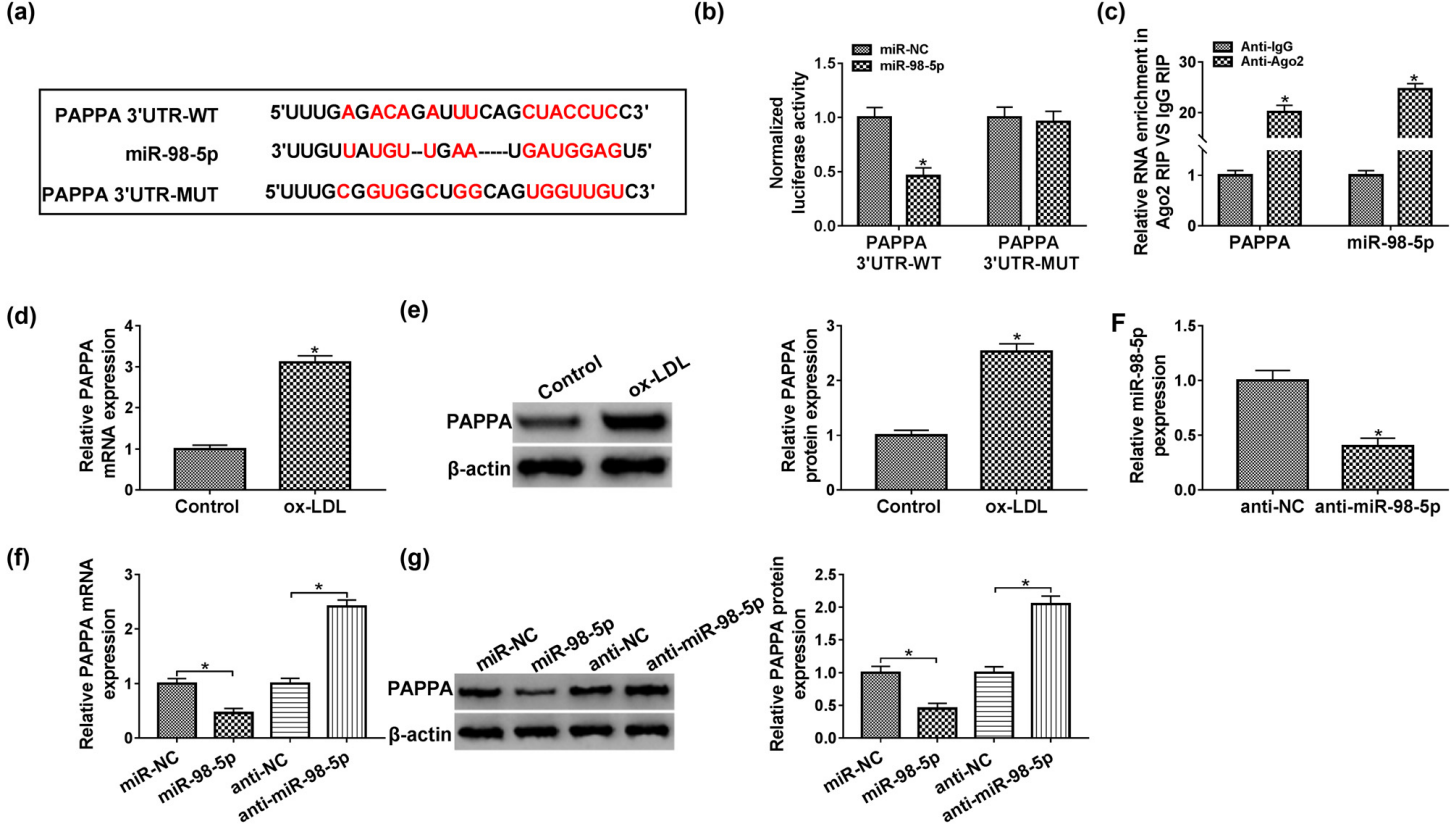
MiR-98-5p negatively regulated PAPPA expression in HUVECs. (a) Binding regions between miR-98-5p and PAPPA, as well as mutated nucleotides, were shown. (b) HUVECs were co-transfected with indicated luciferase reporter vectors and miR-98-5p mimic or miR-NC for dual-luciferase reporter assay. (c) The expression levels of PAPPA and miR-98-5p in Ago2 and IgG group were displayed by RIP assay. (d and e) The mRNA and protein expression levels of PAPPA were assessed by qPCR and western blot assay in HUVECs exposed to ox-LDL, respectively. (f) The expression level of miR-98-5p in HUVECs transfected with anti-NC or anti-miR-98-5p was evaluated by qPCR assay. (g and h) QPCR and western blot assays were applied to measure PAPPA levels in HUVECs transfected with miR-NC, miR-98-5p, anti-NC, or anti-miR-98-5p. Data shown are mean ± SD and from three independent experiments. *P < 0.05. Abbreviations: pregnancy-associated plasma protein A (PAPPA).

The bioinformatics database analysis suggested that miR-98-5p had the putative binding region in 3ʹUTR of PAPPA (Figure 5a). The results of dual-luciferase reporter assay indicated that elevated miR-98-5p could decrease the luciferase activity of PAPPA 3ʹUTR-WT, while luciferase activity of PAPPA 3ʹUTR-MUT was not changed by miR-98-5p in HUVECs (Figure 5b). Additionally, RIP analysis further verified the specificity of interaction relationship between Ago2 and PAPPA mRNA or miR-98-5p in HUVECs (Figure 5c). More importantly, PAPPA was upregulated in HUVECs exposed to ox-LDL (Figure 5d and e). We also found that miR-98-5p was decreased in HUVECs after transfection with anti-miR-98-5p (Figure 5f). Moreover, when compared to the control group, the PAPPA expression was suppressed in the miR-98-5p-overexpressing HUVECs, while PAPPA was upregulated by silencing of miR-98-5p in HUVECs (Figure 5g and h). In summary, miR-98-5p negatively regulated PAPPA expression in HUVECs.

### 3.6 Under ox-LDL condition, knockdown of PAPPA-induced effects on HUVECs could be abolished by silencing of miR-98-5p

**Figure 6:**
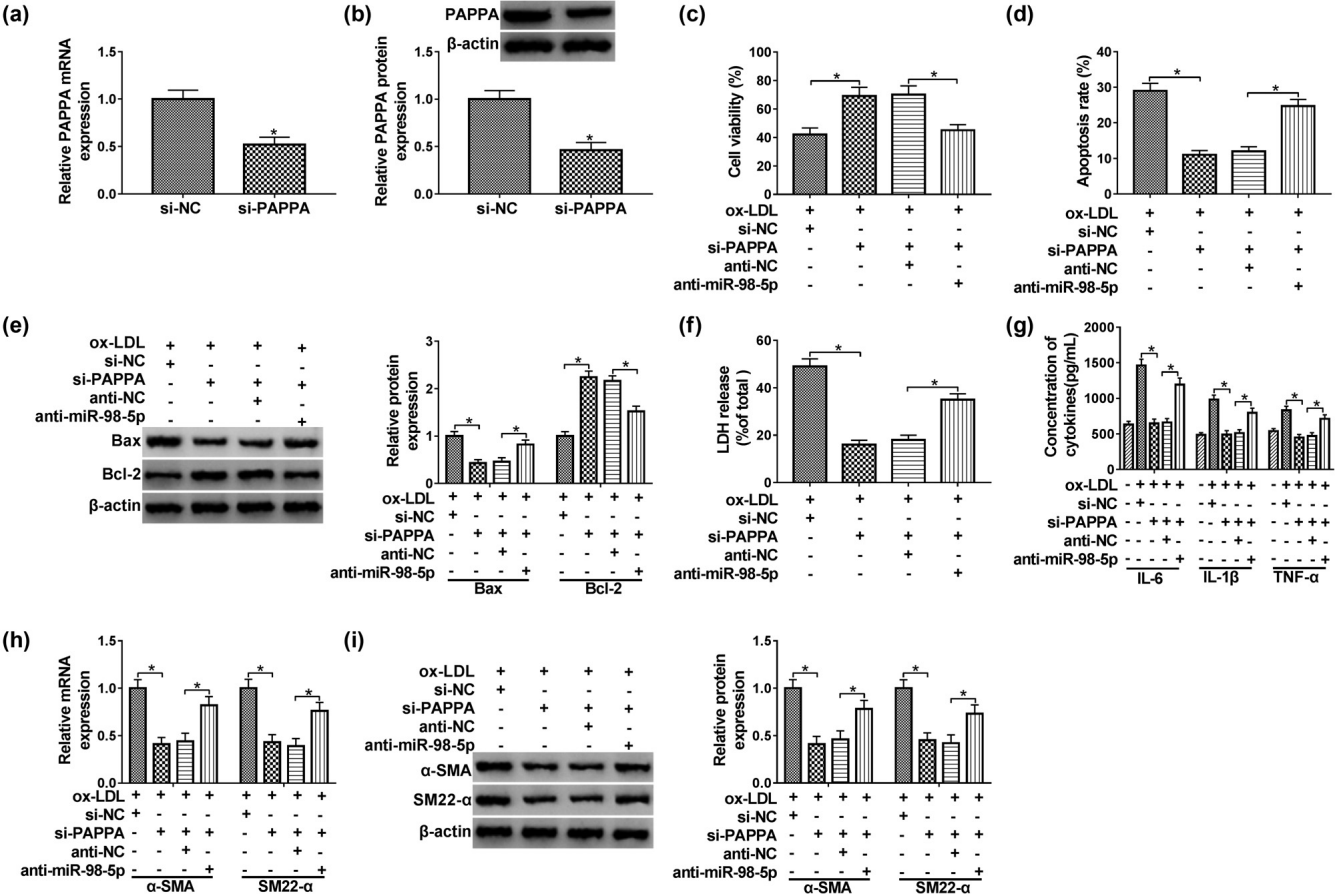
Knockdown of miR-98-5p reversed si-PAPPA induced the effect on proliferation, apoptosis, and inflammatory response in HUVECs. (a and b) The interference efficiency of si-PAPPA in HUVECs was measured using qPCR and western blot assays. (c–f) HUVECs were treated with ox-LDL + si-NC, ox-LDL + si-PAPPA, ox-LDL + si-PAPPA + anti-NC, or ox-LDL + si-PAPPA + anti-miR-98-5p. (c) The cell viability of HUVECs was estimated by MTT assay. (d) Apoptosis analysis was performed in HUVECs using flow cytometry assay. (e) The western blot assay was carried out to analyze Bax and Bcl-2 expression in HUVECs. (f) The LDH detection kit was used to test LDH release in HUVECs. (g) ELISA assay was applied to measure the levels of IL-6, IL-1β, and TNF-α in the medium, with 0 μg/mL of ox-LDL group as control. (h and i) The expression levels of α-SMA and SM22-α in HUVECs were assessed by qPCR and western blot assays. Data shown are mean ± SD and from three independent experiments. *P < 0.05.

The interference efficiency of si-PAPPA was checked by qPCR analysis, and PAPPA was significantly decreased in si-PAPPA group than that in si-NC group (Figure 6a and b). MTT assay revealed that cell viability was dramatically upregulated by transfection with si-PAPPA, which was partly weakened by transfection of anti-miR-98-5p into ox-LDL-induced HUVECs (Figure 6c). Downregulation of PAPPA significantly repressed cell apoptosis, whereas knockdown of miR-98-5p attenuated this change in HUVECs exposed to ox-LDL (Figure 6d). As presented in Figure 6e, silencing of PAPPA inhibited Bax, while increased Bcl-2 expression in HUVECs treated with ox-LDL, which was inverted by transfection of miR-98-5p inhibitor into HUVECs. Besides, LDH release in si-PAPPA + anti-miR-98-5p group was higher compared with si-PAPPA group, indicating that silencing of miR-98-5p promoted LDH release in ox-LDL-induced HUVECs by upregulating PAPPA expression (Figure 6f). ELISA analysis revealed that knockdown of PAPPA led to downregulation of inflammatory factors including IL-6, IL-1β, and TNF-α, which was reversed by silencing of miR-98-5p in ox-LDL-induced HUVECs (Figure 6g). It was shown that the mRNA and protein levels of α-SMA and SM22-α were obviously inhibited in the si-PAPPA group compared with si-NC group, while the si-PAPPA + anti-miR-98-5p group showed the augmentation of α-SMA and SM22-α expression under ox-LDL condition (Figure 6h and i). Synthetically, above data suggested that ox-LDL regulated proliferation, apoptosis, and inflammatory response in HUVECs by targeting miR-98-5p/PAPPA axis.

### 3.7 XIST increased PAPPA expression by regulating miR-98-5p

**Figure 7:**
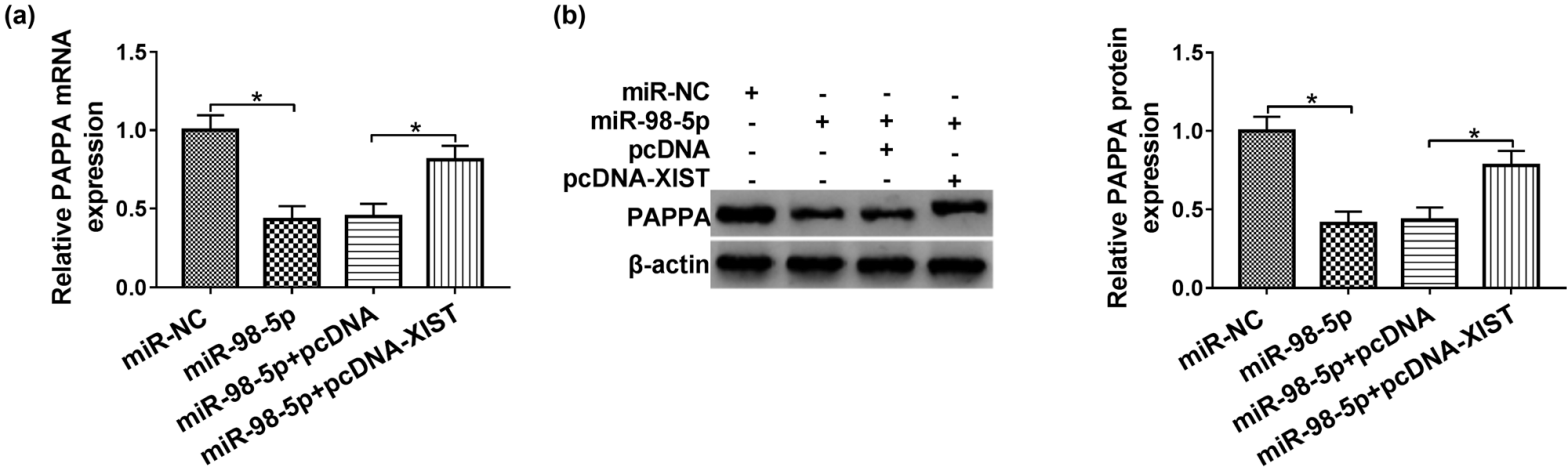
The expression level of PAPPA in HUVECs. (a and b) The mRNA and protein expression levels of PAPPA in HUVECs transfected with miR-NC, miR-98-5p, miR-98-5p + pcDNA, or miR-98-5p + pcDNA-XIST were examined by qPCR and western blot assays, respectively. Data shown are mean ± SD and from three independent experiments. *P < 0.05.

As shown in Figure 7a and b, the results of qPCR and western blot analyses presented that overexpression of miR-98-5p strikingly declined the expression of PAPPA, while this inhibition was abolished by transfecting with pcDNA-XIST into HUVECs. Therefore, PAPPA was regulated by XIST/miR-98-5p axis in HUVECs.

## 4 Discussion

Currently, our results suggested that the cell viability of HUVECs was repressed by ox-LDL in dose/time-dependent method. Consistent with previous research findings [21,22], we also found that ox-LDL could increase HUVECs apoptosis and injury. As we know, HUVECs apoptosis is the key event in the pathogenesis of atherosclerosis [22].

A previous report implied that XIST played a key role in endothelial cell dysfunction [23]. Currently, XIST was overexpressed in HUVECs treated with ox-LDL. Silencing of XIST enhanced proliferation and impeded apoptosis of HUVECs under ox-LDL condition. Similarly, Gu et al. revealed that XIST knockdown protected neuronal cells against spinal cord injury-induced apoptosis [24]. Therefore, XIST may be a key regulator of survival and apoptosis of endothelial cells.

According to a competing endogenous mechanism, lncRNAs can counteract the role of miRNAs by acting as a sponge for miRNAs [25]. For instance, XIST functioned as a molecular sponge to repress the function of miR-101 in gastric cancer [26]. Our results discovered that miR-98-5p was negatively regulated by XIST and was closely associated with apoptosis, proliferation, and inflammation of ox-LDL-stimulated HUVECs. Mechanistically, XIST elevated the PAPPA level by targeting miR-98-5p, which aggravated ox-LDL-induced apoptosis and inflammatory response in HUVECs.

MiRNAs could control gene signaling cascades at post-transcription by binding to target sequences in multiple mRNAs, thereby inducing mRNAs degradation or translational inhibition [27]. MiR-98 has been reported to be connected with cell apoptosis [28]. Chen et al. revealed that miR-98 enhanced the cell growth and extenuated apoptosis of HUVECs exposed to ox-LDL by targetedly inhibiting lectin-like oxidized low-density lipoprotein receptor 1 [29]. As we expected, upregulation of miR-98-5p impeded apoptosis and inflammatory response, as well as upregulated cell viability in HUVECs exposed to ox-LDL; interestingly, upregulation of XIST overturned those effects. Based on these results, miR-98-5p may act as protective roles in vascular disease.

By carrying out bioinformatics database assay and mechanism experiments, PAPPA was discovered as a functional target of miR-98-5p in ox-LDL-induced HUVECs. In HUVECs, PAPPA induced transcription of tissue factor-mRNA, which may result in formation of intracoronary thrombi and endothelial injury [30,31]. Zhang et al. reported that miR-141 inhibited VSMCs proliferation by targeting PAPPA, implying PAPPA might play a momentous part in the pathogenesis of atherosclerosis [32]. In addition, Yang et al. confirmed that PAPPA was upregulated in human vascular smooth muscle cells under ox-LDL administration [33]. In this study, PAPPA was elevated in HUVECs by treatment with ox-LDL. Importantly, silencing of PAPPA-induced inhibitory effects on apoptosis and inflammatory response were abolished by transfection with miR-98-5p inhibitor. PAPPA was proposed as biomarkers and therapeutic targets for atherosclerosis according to our results.

In a nutshell, the data of our study revealed that downregulation of XIST abolished the ox-LDL-induced effects on HUVECs. Functional experiments provided insight into the function of the XIST/miR-98-5p/PAPPA axis in ox-LDL-mediated proliferation, apoptosis, and inflammatory response of HUVECs, which may imply a new molecular biomarker for atherosclerosis.

## 5 Conclusion

In summary, we found that cell proliferation was inhibited, and inflammation and apoptosis were enhanced in HUVECs by treatment with ox-LDL. Furthermore, XIST was upregulated by ox-LDL in HUVECs. Silencing of XIST increased the cell viability and declined cell apoptosis and inflammation reaction in ox-LDL-stimulated HUVECs. Mechanistically, XIST could directly interact with miR-98-5p, and subsequently acted as a miRNA sponge to regulate the expression of PAPPA, which promoted ox-LDL-induced apoptosis, inflammation, and anti-proliferation in HUVECs.
